# Report of a Case of Congenital Bony Occlusion of the Anterior Nares

**Published:** 1888-09

**Authors:** Frank Hamilton Potter

**Affiliations:** Lecturer on Laryngology, Medical Department, Niagara University, Buffalo, N. Y.; No. 273 Franklin Street


### *REPORT OF A CASE OF CONGENITAL BONY OCCLUSION OF THE ANTERIOR NARES.*

By FRANK HAMILTON POTTER, M. D.,

Lecturer on Laryngology, Medical Department, Niagara University, Buffalo, N. Y.

Congenital occlusion of the anterior nares is an extremely rare condition, as an examination of the literature of the subject will show. Two cases have been reported by Dr. W. C. Jarvis*, of New York. In reporting these cases, Dr. Jarvis stated that he had made diligent search for records of other cases without success. The nearest he came to it was a post-mortem case related by Delstanche. This case was unilateral, the right nostril only being imperforate. Dr. Jarvis' cases were symmetrical, both nostrils being occluded. The ages of these cases were eighteen and sixteen years respectively. The case reported below is

* Trans. American Laryn. Ass'n, 1887.

interesting beyond the great rarity of these malformations, from the early discovery of the condition and the success attending the treatment for its removal.

About the first of June, 1888, I was invited by Dr. F. A. Harrington, of this city, to see a child having nasal stenosis, with a view to its relief. The little patient was two years and three months old, of healthy parentage and well developed. When the child was three weeks old, the mother's attention was attracted by its noisy breathing and continually open mouth. Upon looking into the nose, she found the left nostril closed, just within the orifice. As the child grew, it was noticed that she would dig at that side of her nose as if to get something away. The family physician at that time advised the parents to wait until the child was four or five years old before allowing any attempt to be made to remove the obstruction. However, as she became older, the breathing grew more noisy, and they determined to have something done at once. Dr. Harrington was, therefore, consulted, as above stated. Upon investigation it was found, as already intimated, that the family history was very good; in fact, there was nothing of moment in it to be recorded. The child's personal history was also good. She had had a slight stomatitis, which disappeared under treatment; had also had measles with perfect recovery; she was well developed and the upper air passages free from any sign of inflammation; the right nostril was normal in every respect. Upon inspection of the left nostril, a cup-shaped depression was seen about three-eighths of an inch within the orifice; it was glistening-white in appearance and without any opening to the interior of the nose. Of course it was impossible to learn how far the obstruction extended, as no posterior rhinoscopic examination could be made.

An operation was agreed upon and this was performed on the twentieth of June at my office. The child was placed under chloroform by Dr. Harrington, and in order to ascertain the quality of the obstruction a small, strong trochar was pressed firmly against it. The direction of the nasal cavity was kept well in mind by the introduction into the right nostril of a probe, carefully wrapped with cotton, and all instruments used in the left nostril kept parallel with the probe. The obstruction yielded to steady pressure, giving the sensation as if the trochar were passing through bony tissue. After passing in this way fully five-eighths of an inch, it moved with more freedom and was withdrawn. The air immediately rushed into the opening, as was shown by the movement of the *ala* on that side, and the greater ease with which the child breathed, proving the obstruction had been fully pierced. A small galvano-cautery knife under red heat was then introduced, and the opening enlarged to the size of the right nostril as near as could be ascertained. The nostril was finally plugged by cotton, saturated with a four per cent. solution of menthol in *oleum petrolina.* The after-treatment consisted in keeping the parts clean and placing within the nostril a fresh tampon every two to three days, saturated with the same solution.

She made a rapid recovery; the breathing is no longer noisy and is nasal in character; there is no desire to attempt to remove something from the nose; the parts have healed perfectly and there is no discharge from them; the calibre, however, of the left nostril is slightly smaller than that of the right, but not sufficient to warrant any further interference.

But little comment is necessary in this case. Attention, however, is called to the advantage of an early operation. Other malformations of children are relieved by operative measures at even an earlier age than this child, and it is probable from the character of the obstruction that had it been allowed to remain for some years longer, it would have become denser and firmer and much more difficult to remove. It is also probable that, in time, some serious trouble would have resulted from the mouth-breathing and the collecting of the discharges behind the obstruction.

No. 273 Franklin Street.

